# *N*-(3,4-Dichloro­phen­yl)thio­urea

**DOI:** 10.1107/S1600536809035569

**Published:** 2009-09-09

**Authors:** Hai-Bo Shi, Wei-Xiao Hu, Yan-Fang Lin

**Affiliations:** a College of Pharmaceutical Science, Zhejiang University of Technology, Hangzhou 310032, People’s Republic of China; b Zhejiang Pharmaceutical College, Ningbo 315100, People’s Republic of China

## Abstract

In the title compound, C_7_H_6_Cl_2_N_2_S, the benzene ring and the mean plane of the thio­urea fragment [—N—C(=S)—N] make a dihedral angle of 66.77 (3)°. Inter­molecular N—H⋯S and N—H⋯Cl hydrogen bonds link the mol­ecules into a three-dimensional network.

## Related literature

For the synthesis of the title compound, see: Liu *et al.* (1994 ▶). For details of the biological activity of thia­zole and its derivatives, see: Holla *et al.* (2003 ▶).
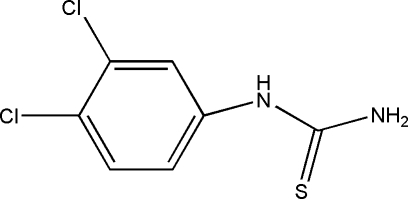


## Experimental

### 

#### Crystal data

C_7_H_6_Cl_2_N_2_S
*M* *_r_* = 221.10
Triclinic, 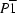

*a* = 5.8168 (19) Å
*b* = 8.489 (3) Å
*c* = 9.771 (3) Å
α = 107.042 (4)°
β = 94.468 (4)°
γ = 94.778 (4)°
*V* = 457.0 (3) Å^3^
*Z* = 2
Mo *K*α radiation
μ = 0.88 mm^−1^
*T* = 291 K
0.15 × 0.10 × 0.08 mm

#### Data collection

Bruker SMART APEX CCD area-detector diffractometer
Absorption correction: multi-scan (*SADABS*; Sheldrick, 1996 ▶) *T*_min_ = 0.879, *T*_max_ = 0.933
1882 measured reflections
1562 independent reflections
1410 reflections with *I* > 2σ(*I*)
*R*_int_ = 0.062

#### Refinement

*R*[*F* ^2^ > 2σ(*F* ^2^)] = 0.084
*wR*(*F* ^2^) = 0.236
*S* = 1.10
1562 reflections
122 parameters
3 restraints
H atoms treated by a mixture of independent and constrained refinement
Δρ_max_ = 0.75 e Å^−3^
Δρ_min_ = −0.76 e Å^−3^

### 

Data collection: *SMART* (Bruker, 2005 ▶); cell refinement: *SAINT* (Bruker, 2005 ▶); data reduction: *SAINT*; program(s) used to solve structure: *SHELXS97* (Sheldrick, 2008 ▶); program(s) used to refine structure: *SHELXL97* (Sheldrick, 2008 ▶); molecular graphics: *SHELXTL* (Sheldrick, 2008 ▶); software used to prepare material for publication: *SHELXTL*.

## Supplementary Material

Crystal structure: contains datablocks global, I. DOI: 10.1107/S1600536809035569/bg2297sup1.cif

Structure factors: contains datablocks I. DOI: 10.1107/S1600536809035569/bg2297Isup2.hkl

Additional supplementary materials:  crystallographic information; 3D view; checkCIF report

## Figures and Tables

**Table 1:** Hydrogen-bond geometry (Å, °)

*D*—H⋯*A*	*D*—H	H⋯*A*	*D*⋯*A*	*D*—H⋯*A*
N7—H7*X*⋯S9^i^	0.86 (3)	2.51 (2)	3.342 (3)	161 (4)
N10—H10*Y*⋯Cl1^ii^	0.87 (3)	2.80 (2)	3.646 (3)	163 (4)

Symmetry codes: (i) 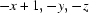; (ii) 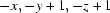.

**Fig. 1.**
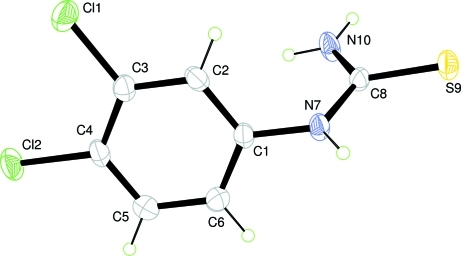
The structure of (I), shown with 30% probability displacement ellipsoids.

**Fig. 2.**
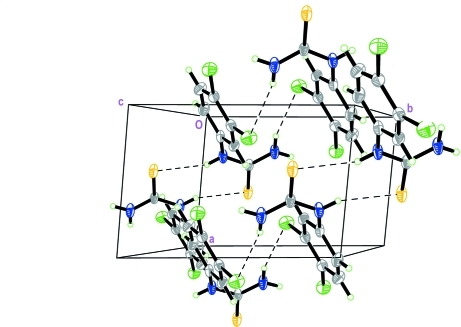
N—H···S and N—H···Cl interactions (dotted line) in the title compound.

## supplementary crystallographic information

### Comment

Thiazoles and their derivatives are found to be associated with various biological activities such as antibacterial, antifungal, anti-inflammatory activities(Holla *et al.*, 2003).The title compound, *N*-(3,4-dichlorophenyl)thiourea(I),is an important intermediate in the synthesis of thiazole and their derivatives. In our work, we present its crystal structure. In Fig.1, the benzene ring of (I) is twisted out ofthe mean plane through the –N7—C8(=S9)—N10 group by a diherdral angle of 66.77 (3)°. Weak intermolecular N—H···S and N—H···Cl hydrogen bonds (Table 1) link the molecules into a three-dimensional network.

### Experimental

The title compound was obtained by refluxing 3,4-dichloroaniline(48.6 g, 0.3 mol), 36% aqueous HCl(30.4 g,0.3 mol) and ammonium thiocyanate(22.8 g, 0.3 mol) in water for 7 hr, then a white precipitate was observed and filtered. The solid was recrystallized from alcohol to give the pure product. This was dissolved in THF, and the solution evaporated gradually at room temperature to afford single crystals of (I).(m.p. 489–490 K). MS(m/*z*,%): 220 (*M*^+^, 90), 187 (15), 178 (16), 161 (98), 126 (7), 99 (10), 74 (8), 60 (55).

### Refinement

Atoms H7X, H10X and H10Y were located in difference Fourier maps and refined isotropically with the N—H bond restraint of 0.87 (2) Å. Other H atoms were placed in calculated positions with C—H = 0.93 Å, and refined in riding mode, with *U*_iso_(H) = 1.2 *U*_eq_(C).

### Crystal data

**Table:**

C_7_H_6_Cl_2_N_2_S	*Z* = 2
*M**_r_* = 221.10	*F*(000) = 224
Triclinic, *P*1	*D*_x_ = 1.607 Mg m^−^^3^
Hall symbol: -P 1	Mo *K*α radiation, λ = 0.71073 Å
*a* = 5.8168 (19) Å	Cell parameters from 843 reflections
*b* = 8.489 (3) Å	θ = 2.5–27.0°
*c* = 9.771 (3) Å	µ = 0.88 mm^−^^1^
α = 107.042 (4)°	*T* = 291 K
β = 94.468 (4)°	Prism, orange
γ = 94.778 (4)°	0.15 × 0.10 × 0.08 mm
*V* = 457.0 (3) Å^3^	

### Data collection

**Table:**

Bruker SMART APEX CCD area-detector diffractometer	1562 independent reflections
Radiation source: fine-focus sealed tube	1410 reflections with *I* > 2σ(*I*)
graphite	*R*_int_ = 0.062
φ and ω scans	θ_max_ = 25.0°, θ_min_ = 2.2°
Absorption correction: multi-scan (*SADABS*; Sheldrick, 1996)	*h* = −6→3
*T*_min_ = 0.879, *T*_max_ = 0.933	*k* = −9→10
1882 measured reflections	*l* = −11→11

### Refinement

**Table:**

Refinement on *F*^2^	Secondary atom site location: difference Fourier map
Least-squares matrix: full	Hydrogen site location: inferred from neighbouring sites
*R*[*F*^2^ > 2σ(*F*^2^)] = 0.084	H atoms treated by a mixture of independent and constrained refinement
*wR*(*F*^2^) = 0.236	*w* = 1/[σ^2^(*F*_o_^2^) + (0.1955*P*)^2^] where *P* = (*F*_o_^2^ + 2*F*_c_^2^)/3
*S* = 1.10	(Δ/σ)_max_ < 0.001
1562 reflections	Δρ_max_ = 0.75 e Å^−^^3^
122 parameters	Δρ_min_ = −0.76 e Å^−^^3^
3 restraints	Extinction correction: *SHELXL97* (Sheldrick, 2008), Fc^*^=kFc[1+0.001xFc^2^λ^3^/sin(2θ)]^-1/4^
Primary atom site location: structure-invariant direct methods	Extinction coefficient: 0.13 (4)

### Special details

**Table:**

Geometry. All e.s.d.'s (except the e.s.d. in the dihedral angle between two l.s. planes) are estimated using the full covariance matrix. The cell e.s.d.'s are taken into account individually in the estimation of e.s.d.'s in distances, angles and torsion angles; correlations between e.s.d.'s in cell parameters are only used when they are defined by crystal symmetry. An approximate (isotropic) treatment of cell e.s.d.'s is used for estimating e.s.d.'s involving l.s. planes.
Refinement. Refinement of *F*^2^ against ALL reflections. The weighted *R*-factor *wR* and goodness of fit *S* are based on *F*^2^, conventional *R*-factors *R* are based on *F*, with *F* set to zero for negative *F*^2^. The threshold expression of *F*^2^ > σ(*F*^2^) is used only for calculating *R*-factors(gt) *etc*. and is not relevant to the choice of reflections for refinement. *R*-factors based on *F*^2^ are statistically about twice as large as those based on *F*, and *R*- factors based on ALL data will be even larger.

### Fractional atomic coordinates and isotropic or equivalent isotropic displacement parameters (Å^2^)

**Table:**

	*x*	*y*	*z*	*U*_iso_*/*U*_eq_	
Cl1	0.19736 (17)	0.43998 (14)	0.66939 (11)	0.0560 (6)	
Cl2	−0.27770 (17)	0.21189 (14)	0.62155 (11)	0.0565 (6)	
C1	0.1662 (6)	0.1685 (4)	0.2584 (4)	0.0365 (9)	
C2	0.2383 (6)	0.2836 (4)	0.3921 (4)	0.0398 (9)	
H2	0.3777	0.3512	0.4062	0.048*	
C3	0.1023 (6)	0.2971 (4)	0.5034 (4)	0.0374 (9)	
C4	−0.1046 (6)	0.1948 (4)	0.4830 (4)	0.0388 (9)	
C5	−0.1748 (6)	0.0795 (5)	0.3507 (4)	0.0480 (11)	
H5	−0.3127	0.0103	0.3372	0.058*	
C6	−0.0398 (7)	0.0669 (4)	0.2380 (4)	0.0446 (9)	
H6	−0.0879	−0.0101	0.1485	0.054*	
N7	0.3073 (6)	0.1521 (3)	0.1444 (3)	0.0424 (9)	
H7X	0.371 (7)	0.061 (4)	0.113 (5)	0.071 (15)*	
C8	0.3732 (6)	0.2709 (4)	0.0853 (4)	0.0353 (8)	
S9	0.58001 (18)	0.24215 (10)	−0.03037 (11)	0.0493 (6)	
N10	0.2718 (6)	0.4075 (4)	0.1191 (4)	0.0508 (10)	
H10X	0.308 (7)	0.486 (5)	0.083 (5)	0.055 (12)*	
H10Y	0.159 (5)	0.422 (6)	0.173 (4)	0.054 (12)*	

### Atomic displacement parameters (Å^2^)

**Table:**

	*U*^11^	*U*^22^	*U*^33^	*U*^12^	*U*^13^	*U*^23^
Cl1	0.0546 (8)	0.0620 (8)	0.0427 (7)	0.0057 (5)	0.0146 (5)	0.0001 (5)
Cl2	0.0577 (8)	0.0636 (9)	0.0549 (8)	0.0087 (5)	0.0328 (5)	0.0209 (6)
C1	0.0534 (19)	0.0253 (16)	0.0377 (19)	0.0123 (14)	0.0214 (15)	0.0137 (14)
C2	0.0404 (18)	0.0334 (18)	0.050 (2)	0.0026 (14)	0.0167 (15)	0.0160 (16)
C3	0.0453 (19)	0.0341 (18)	0.0375 (19)	0.0122 (14)	0.0132 (14)	0.0139 (15)
C4	0.0433 (19)	0.0396 (19)	0.042 (2)	0.0106 (15)	0.0200 (15)	0.0195 (16)
C5	0.048 (2)	0.043 (2)	0.051 (2)	−0.0046 (16)	0.0107 (18)	0.0127 (18)
C6	0.060 (2)	0.0344 (19)	0.039 (2)	0.0023 (15)	0.0123 (16)	0.0087 (15)
N7	0.064 (2)	0.0251 (15)	0.0460 (18)	0.0146 (13)	0.0319 (14)	0.0136 (13)
C8	0.0489 (19)	0.0257 (16)	0.0338 (18)	0.0059 (13)	0.0167 (14)	0.0091 (13)
S9	0.0722 (9)	0.0288 (7)	0.0572 (8)	0.0151 (5)	0.0417 (6)	0.0175 (5)
N10	0.072 (2)	0.0312 (17)	0.064 (2)	0.0175 (15)	0.0434 (17)	0.0239 (15)

### Geometric parameters (Å, °)

**Table:**

Cl1—C3	1.733 (4)	C5—C6	1.386 (5)
Cl2—C4	1.729 (4)	C5—H5	0.9300
C1—C6	1.382 (5)	C6—H6	0.9300
C1—C2	1.391 (5)	N7—C8	1.345 (4)
C1—N7	1.416 (5)	N7—H7X	0.87 (2)
C2—C3	1.378 (6)	C8—N10	1.312 (5)
C2—H2	0.9300	C8—S9	1.698 (4)
C3—C4	1.389 (5)	N10—H10X	0.86 (3)
C4—C5	1.380 (6)	N10—H10Y	0.87 (3)
			
C6—C1—C2	120.1 (3)	C6—C5—H5	120.0
C6—C1—N7	120.0 (3)	C1—C6—C5	120.0 (3)
C2—C1—N7	120.0 (3)	C1—C6—H6	120.0
C3—C2—C1	119.7 (3)	C5—C6—H6	120.0
C3—C2—H2	120.1	C8—N7—C1	126.3 (3)
C1—C2—H2	120.1	C8—N7—H7X	114 (3)
C2—C3—C4	120.2 (3)	C1—N7—H7X	119 (3)
C2—C3—Cl1	118.9 (3)	N10—C8—N7	118.0 (3)
C4—C3—Cl1	120.9 (3)	N10—C8—S9	121.7 (3)
C5—C4—C3	119.9 (3)	N7—C8—S9	120.4 (3)
C5—C4—Cl2	119.5 (3)	C8—N10—H10X	121 (3)
C3—C4—Cl2	120.6 (3)	C8—N10—H10Y	123 (3)
C4—C5—C6	120.0 (3)	H10X—N10—H10Y	116 (4)
C4—C5—H5	120.0		
			
C6—C1—C2—C3	−0.7 (5)	Cl2—C4—C5—C6	178.7 (3)
N7—C1—C2—C3	−178.9 (3)	C2—C1—C6—C5	0.0 (5)
C1—C2—C3—C4	0.9 (5)	N7—C1—C6—C5	178.1 (3)
C1—C2—C3—Cl1	179.3 (3)	C4—C5—C6—C1	0.6 (5)
C2—C3—C4—C5	−0.2 (5)	C6—C1—N7—C8	121.2 (4)
Cl1—C3—C4—C5	−178.7 (3)	C2—C1—N7—C8	−60.7 (5)
C2—C3—C4—Cl2	−179.5 (2)	C1—N7—C8—N10	−11.2 (5)
Cl1—C3—C4—Cl2	2.1 (4)	C1—N7—C8—S9	169.3 (3)
C3—C4—C5—C6	−0.5 (5)		

### Hydrogen-bond geometry (Å, °)

**Table:**

*D*—H···*A*	*D*—H	H···*A*	*D*···*A*	*D*—H···*A*
N7—H7X···S9^i^	0.86 (3)	2.51 (2)	3.342 (3)	161 (4)
N10—H10Y···Cl1^ii^	0.87 (3)	2.80 (2)	3.646 (3)	163 (4)

Symmetry codes: (i) −*x*+1, −*y*, −*z*; (ii) −*x*, −*y*+1, −*z*+1.
